# Evolution of Pediatric Migraine Patients Admitted at an Emergency Department after a 10-Year Follow-Up

**DOI:** 10.3390/jcm12072475

**Published:** 2023-03-24

**Authors:** Maria Laura Manzo, Federica Reina, Edvige Correnti, Francesca D’Aiuto, Daniela D’Agnano, Andrea Santangelo, Luigi Vetri, Giuseppe Santangelo, Laura Maniscalco, Gabriele Tripi, Vittorio Sciruicchio, Vincenzo Raieli

**Affiliations:** 1 Child Neuropsychiatry Unit Department, Pro.M.I.S.E. “G D’Alessandro”, University of Palermo, 90100 Palermo, Italy; 2 Child Neuropsychiatry Department, ISMEP-ARNAS Civico, 90100 Palermo, Italy; 3 Emergency Department, ISMEP-ARNAS Civico, 90100 Palermo, Italy; 4 Children Epilepsy and EEG Center, PO, San Paolo ASL, 70132 Bari, Italy; 5 Pediatrics Department, AOUP Santa Chiara Hospital, 56126 Pisa, Italy; 6 Oasi Research Institute IRCCS, 94018 Troina, Italy

**Keywords:** migraine, children, follow-up, emergency department, headache, sleep, headache

## Abstract

Background: Despite its high prevalence, the clinical course of pediatric migraine has not been fully understood, and previous studies present conflicting results. We present here the findings of a 10-year follow-up study involving children with severe migraine pain admitted to our emergency department. Furthermore, all studies were carried out on selected outpatient clinical case studies. Our aim was to evaluate a population of migraine children admitted to an emergency department because of increased severity or frequency of pain or even because of very anxious parents concerning their child’s headache in order to describe their long-term outcomes, whether it differed from that of outpatient populations and to identify possible predictors of prognosis. Methods: We recruited 80 subjects with migraine headaches (mean age 8 years with a range of 4–14 years, 50% females), attending the baseline examination of a population admitted for a headache to the Emergency Department in the first half year of 2012. Of the 80 subjects, 48 (60%) were eligible for follow-up in 2022. We included in our study only patients diagnosed with migraine, according to the diagnostic criteria of the International Classification of Headache Disorders. All were contacted by telephone, and a semi-structured questionnaire was provided to them by email. The association between several possible prognostic factors (gender, familiar neurologic disorders, prenatal and perinatal disorders, social activities, sleep disorders, etc.) and the long-term persistence of migraine headaches were explored using logistic regression analysis. Results: Of 48 subjects with migraine headaches at baseline, 31 (65%) had persistent migraine, and 17 (35%) experienced remission. The preliminary results showed that the presence of neurologic disorders in parents (*p* < 0.01—odds ratio 9.34 (2.53–41.64) and sleep disorders (*p* < 0.01—odds ratio 13.18 (2.25–252.74) significantly predicted the 10-year persistence of migraine headaches, while the other considered predictors were found not to influence prognosis. Conclusions: To our knowledge, this was the first study conducted on a selected pediatric population upon admission to the emergency room. Our study suggests that a population of pediatric migraine selected for admission to the emergency department also shows a favorable long-term prognosis, like the studies conducted in the outpatient sample. Familial neurological comorbidity and sleep disorders were unfavorable factors for predicting good outcomes.

## 1. Introduction

Headache is a very common neurological cause of admission to the Pediatric Emergency Department, accounting for 1–4% of visits [1]. Due to their often disabling nature, headaches are a health problem of considerable importance that induce a lot of fear in children and their caregivers, for both the actual pain and for the children’s future about their headaches [2]. The frequency and urgency perceived of headache attacks are such to determine an increasingly frequent use of the services of the emergency department [3,4]. Among primary pediatric headaches, migraine is certainly the main one in terms of disability and admission to the emergency department. It is a recurrent headache characterized by severe pain in intensity that forces interruptions in activities, usually lasting more than 1 h in children, associated with general neurovegetative disorders, phonophobia, and often transient neurological disorders (aura). In children, the pain is often bilateral and associated with allodynia and cranial autonomic symptoms (lacrimation, rhinorrhoea etc.). The short and long-term outcome of primary headaches, particularly migraine, in childhood has been evaluated in various studies [5]. Further studies were conducted on patients followed in the outpatient clinic and not on patients accessing an emergency department, where patients generally tend to go because of more severe migraine attacks [5,6,7,8,9]. Therefore, we wondered if this aspect could influence the long-term prognosis. We, therefore, initiated a long-term follow-up study on children and adolescents who were referred to the emergency department for moderate/severe migraine attacks that are difficult to manage at home. The primary objective of this study was to evaluate the long-term prognosis 10 years after the first emergency department access for a moderate/severe migraine attack in a group of children and adolescents with migraine. Another objective is to enlighten eventual predictive factors of a possible greater risk of persistence of migraine.

## 2. Methods

We conducted a 10-year follow-up study by selecting the population of pediatric patients who were admitted to the Emergency Department of the “Di Cristina” Pediatric Hospital for headache attacks in 2012. From the Emergency Department archives, it emerged that in 2012, 250 patients were hospitalized for headaches. We included in our study only patients undergoing a migraine diagnosis in 2012, formulated according to the diagnostic criteria of the International Classification of Headache Disorders (ICHD-2) [10]. All diagnoses were reviewed by child neuropsychiatry staff and a neurologist specializing in headache disorders.

Therefore, the exclusion criteria at inclusion were primary headaches other than migraine (tension-type headaches and trigeminal autonomic headaches), secondary headaches (headaches associated with fever or respiratory infection, head injury, or tumor and vascular disease) and unspecified headaches. The study involved sending a questionnaire by mail, preceded by a brief telephone interview.

The questionnaire is divided into eight sections:

Section I: patient’s personal data including age, gender, educational qualifications of parents, presence of siblings, presence of neurologic disorders in parents and presence of headache.

Section II: information on pregnancy and on adaptation to extrauterine life: gestational age, any perinatal discomforts, birth weight, and age of acquisition of the main neuro psychomotor phases such as autonomous walking, language, and sphincter control.

Section III: general information relating to the patient, such as school attended, academic performance, hours spent on social networks and video games, sports practiced and current weight, sleep disturbances and eating habits.

Section IV: any present or previous comorbidities.

Section V: information about the characteristics of migraine attacks referring to the period in which they entered the Emergency Department in 2012: monthly frequency of attacks, duration, location, pain intensity, possible worsening of headache with or after physical activity and any other symptoms.

Section VI: information about any admission to the Emergency Department under ordinary or Day Service or Day Hospital arrangements. This section also investigates any neuroradiological and/or electrophysiological investigations carried out after admission to the ED.

Section VII: only refers to patients who continue to suffer from migraine in 2022 after 10 years and investigates current headache characteristics, including current monthly frequency of migraine attacks, course, duration, location, intensity, type of pain, time of pain onset during the day, any associated symptoms and therapies, both those taken as needed and as prophylaxis, etc.

Section VIII: explores the characteristics and possible modifications of headaches during the SARS-CoV-2 pandemic.

This study was approved by the Scientific Ethics Committee (the minutes n.51; prot-amm.n.263 Civico 14/1272020).

### Statistical Analysis

Continuous variables were summarized with median and interquartile range (IQR), while categorical variables were expressed as counts and percentages. The change in frequency of attacks/month, duration, intensity, worsening by the movement, and associated symptoms between 2012 and 2022 was assessed by the McNemar exact test for binary matched-pairs data. The associations between several possible prognostic factors (gender, familiar neurological disorders, prenatal and perinatal disorders, social activities, sleep disorders etc.) and the long-term persistence of migraine headaches were explored using logistic regression analysis. The odds ratio of univariable and multivariable logistic regression models were calculated and reported with 95% confidence intervals (95% CI). Statistical analysis was performed with R software (version 4.2.2), and a *p*-value < 0.05 was considered statistically significant.

## 3. Results

**Figure 1:**
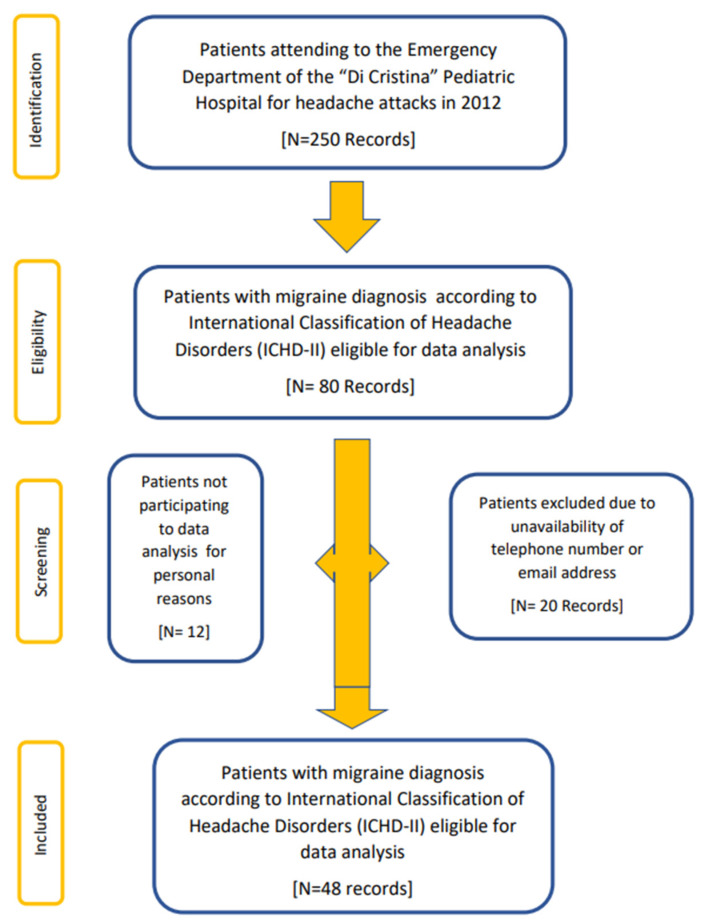
Flow chart diagram of subject selection.

At baseline, 80 patients met the inclusion criteria. However, of these 80, 20 could not be contacted due to the unavailability of the telephone number or email address due to the computerization of medical records, and 12 did not participate in the study for personal reasons. Therefore, 48 patients (60%) were included in the follow-up study; in 2022, 10 years later, they were contacted by telephone, and after a brief informational interview on the type of study, we provided them by email with an anonymous semi-structured questionnaire to be filled in, with full respect of their privacy and only after they gave their informed consent. The flow chart shows the selection of the sample (Figure 1).

In 2012, the median age of the 48 patients who entered the Emergency Department for severe migraine attacks and who joined the study was eight years (IQR = 6–9 years). The frequency distribution shows that the group is made up of 24 males and 24 females; therefore, it is equally distributed with respect to gender (see Table 1).

Our study shows that in 2012 the average frequency of migraine attacks was 2.6 attacks/month, 52% had ≤2 attacks/month and 48% ≥3 attacks/month, while the average duration of the attacks was 2 h 14 min, specifically in 58% of cases it lasted ≤2 h, while in 17% of cases it lasted >2 h (25% did not remember the duration of the pain). Some 88% of the interviewees answered that they had had migraine attacks of severe/unbearable intensity (pain forced them to stop all activity), while 8% had moderate intensity (pain forced them to reduce all activity), and 4% did not remember it well. Of the patients, 66% had all associated symptoms during the migraine attack (vomit, nausea, phono/photophobia), 32% had at least one symptom, and 2% did not remember. Neuroradiological and neurophysiological exams were negative. In 2022, 10 years later, the prevalence of migraine was 65% (31/48), while 35% (17/48) were free from headaches. The average frequency of attacks was 3.1 attacks per month, and 50% had ≤2 attacks/month and 50% ≥3 attacks/month, while the average duration of attacks was 2 h and 6 min, specifically in the 80.5% of cases that lasted ≤2 h, while in 19.5% of cases it lasted >2 h. Of those interviewed, 74% reported continuing to have migraine attacks of severe/unbearable intensity, while 26% had attacks of moderate intensity. After 10 years, 71% of patients reported having all associated symptoms during the migraine attack, while 19% did not have all of them. From the analysis of the data on the location of the pain at the 10-year follow-up, it emerges that 42% had pain sometimes unilaterally and sometimes bilaterally, while 29% had bilateral pain, 19% unilateral pain and 10% had pain at the vertex. In 61.3% of the interviewees, the pain was throbbing. For 16.1%, the pain was constricting, while for 19.4%, it was pressing, and for 3.2%, it was stabbing. Eighty percent of the 31 patients interviewed who continue to suffer from migraine after 10 years reported taking medications as needed (analgesics and/or anti-inflammatories), while 10% take antiepileptic drugs (topiramate, lamotrigine, sodium valproate) and 10% take triptans (sumatriptan), and 83% reported having had clinical benefit from taking these drugs. By comparing the migraine characteristics of 2012 (n. 48) and 2022 (n. 31), there were no statistically significant differences in terms of attack frequency, duration, aggravation of pain by movements and associated symptoms. However, we observed that the intensity of pain seems to decrease after 10 years, with a *p*-value calculated with the McNemar exact test of 0.038. The migraine features of the sample in 2012 and 2022 are reported and compared in Table 2.

In our study we analyzed the possible long-term prognostic factors (Table 3) and from the univariable analysis, it emerged that the presence of neurologic disorders in the parents (OR: 9.34, 95% CI: 2.53–41.64, *p*-value = 0.001), sleep disorders (OR: 11.29, 95% CI: 1.88–297.28, *p*-value = 0.018), and the frequency of attacks greater than or equal to three in 2012 (OR: 8.48, 95% CI: 2.21–43.00, *p*-value = 0.003) are significantly statistically associated with a worse prognosis and increased risk of chronic migraine (see Table 3). However, in the multivariable analysis, which included only neurological disorders in parents and frequency of attacks/months in 2012, only the presence of neurologic disorders in parents increased the risk of having chronic migraine (OR: 5.28, 95% CI: 1.21–25.83), *p*-value = 0.030) (see Table 4).

## 4. Discussion

To our knowledge, in the literature, there are no follow-up studies of pediatric migraine patients admitted to emergency departments. This would be the first 10-year long-term follow-up study of migraine in pediatric patients admitted to the emergency department for moderate/severe migraine attacks. We wondered if this could affect the long-term prognosis. Our study suggests some interesting points:

(A) migraine in children had a favorable prognosis for attacks that had led them to the emergency department. Therefore, despite hospitalization at the emergency department for migraine attacks of moderate/severe intensity, the percentage (35%) of remission at 10 years in patients admitted to the ED was substantially the same as the percentage of remission of migraine outpatients [9,11,12].

(B) Subjects still suffering from migraine after 10 years did not show an increase in the frequency, similar to the data in the literature. Furthermore, the migraine attacks showed an improvement in the intensity of pain. 

(C) In our study, we analyzed the possible long-term prognostic factors (Table 3), and it emerged that the presence of neurologic disorders in the parents (*p*-value = 0.001), and in particular with headache and/or migraine (48% of patients referred a family history of persistent migraine headache/migraine) and sleep disorders (*p*-value = 0.018) were statistically significantly associated with a worse prognosis and increased risk of chronic migraine.

- Outcome: comparison with literature data

Several studies point out that childhood migraine tends to regress or improve. However, it is not always easy to compare different studies because of the difference in diagnostic criteria used before or after the introduction of the International Classification of Headache Disorders diagnostic criteria [5,6,7,10].

Furthermore, subjective assessment of the current situation or memory bias could reduce the assessment of headache evolution. The short-term and long-term outcome of primary headaches, in particular, migraine in childhood, has been evaluated in various studies. In a five-year follow-up study of a school population of 64 migraineurs (ages 11–14), remission rates were 19%, persistent migraines 65%, episodic tension headaches comprised 13%, and unclassifiable headaches comprised 3% [6]. In another 10-year follow-up study of the same patients, the rates were 38.2% headache-free, 42% with persistent migraine, and 20% progressing to tension-type headache, respectively [12]. In another 10-year follow-up study by Galinski et al. of 84 migraine patients (aged 8–14 years), 14% were headache-free, 46% had persistent migraine, and 40% had type headache tensive [9]. In another eight-year follow-up study of 77 primary headache patients (aged 3 to 16 years), 27.3% were headache-free, 42.9% had persistent migraine, and 28.6% had tension-type headaches [7]. In an eight-year longitudinal study of 100 patients with a mean age of 17.9 years, Guidetti and Galli analyzed the evolution of migraine and tension headaches; they found that migraine was less likely to remit than chronic tension headache (28.1% versus 44.4%) [13]. According to Sillampää, who followed school children with migraine with onset before the age of eight for seven years, migraine regressed for 22% of patients and improved in 37% of cases [14]. According to Brna et al. [15], who conducted a 20-year follow-up study, it appeared that headache remission at short-term follow-up does not necessarily predict long-term resolution. The persistence of headaches at 10 years appears to be more predictive of the persistence of headaches into adulthood, particularly if the primary headache is a migraine. Their study shows that after 10 years of follow-up, 25% of patients were without headaches, 30% had tension headaches, 41% had migraine, and 3% had migraine and tension headaches simultaneously. However, at the 20-year follow-up, it was highlighted that 27% had no headache, 33% had a tension headache, 17% had migraines, and 23% had migraines and tension headaches at the same time [15]. Another very important study is the one conducted in the 1990s by Bille. After following a group of 73 schoolchildren with migraine with onset between the ages of 7 and 13 for 40 years, Bille found that 23% were free from migraines at age 25, and over 50% continued to have migraines at age 50 [11].

To aid better understanding, we compare our data with the main 10-year follow-up studies in Table 5.

It shows that in most cases, the sample size, the rate of loss of subjects at 10 years, the rate of persistent migraine and the rate of headache-free or transformation to another type of headache, the method of investigation for carrying out the study are similar. For these reasons, it can be concluded with reasonable evidence that pediatric migraine has a good long-term prognosis (8–40 years) with periods of long remission or improvement. This finding is found whether studied in a general population [6,12,14], in clinical populations at selected centers [7,8,9,11,13,14,15,16,17,18,19,20,21] or in an emergency department population as in our study.

### 4.1. Prognostic Factors: Comparison with Literature Data

Literature studies show the role of various possible prognostic factors, in some cases with controversial results. The considerable variability of the studies’ results is due to methodological differences regarding the population studied, the diagnostic criteria, the duration of the follow-up and the methods for evaluating the results. Galinski, in his follow-up study, reports that age > 12 years at first presentation was statistically associated with long-term chronic migraine [9]. Among possible prognostic factors, early age of onset [17,18], psychosocial stress factors and psychiatric comorbidity may be related to a poor long-term prognosis, while the role of gender as a prognostic factor still remains unclear and much debated [13]. In our study, the impact of the female gender does not reach the level of statistical significance in the multivariate analysis. Therefore, for us, the female gender does not constitute an unfavorable prognostic factor. However, it should be emphasized that migraine tends to persist more in females (55%) than in males in our sample. Furthermore, the data reported in the literature on the role of gender in the evolution of migraine are sometimes discordant. Some authors, such as Guidetti and Bille in their various studies, reported a poor prognosis in women compared to males [11,12], while other authors, such as Metsähonkala and Mazzotta, reported a poor prognosis in males [19,20]. Other factors associated with a poor prognosis are a family history of migraine, onset before six years of age [18], and attack intensity [8,9,11,15,21]. Recently Orr et al. [22] reported the unfavorable prognosis in youth migraineurs with a history of status migrainosus, in some ways similar to our sample of migraineurs selected for emergency department admissions. However, their follow-up was very short (one to three months) and not comparable to our long-term follow-up. Our study supports a poorer prognosis in the presence of familiarity for migraine. These data are in accordance with those of previous population-based family studies and twin studies, and they suggest the importance of studying the role of genetic factors in migraine, especially in younger children [12,23].

There is a high comorbidity between migraine and sleep disorders. Sleep disturbances are very common. About 25% of children suffer from them [24]. There is a mutual dependence between sleep and headaches. Inadequate or prolonged sleep duration or poor sleep quality are triggers of migraine attacks, and frequent nocturnal awakenings precede sleep-related migraine. In addition, poor sleep has a correlated state of migraine chronicity. On the other hand, migraine can be seen as a possible cause of sleep disturbances [24,25,26].

### 4.2. Limitations

Our study has some major limitations: the sample size was small at baseline, especially considering the likely loss rate at a 10-year follow-up. However, previous studies have also had similar sample sizes. See Table 5 [8,9,12]. Also, the relative loss of patients at follow-up (40%) is within the range of 22% to 44% described and published in the literature [27]. The general characteristics of people lost at the follow-up were not significantly different from those of our interviewed patients. Furthermore, our study has the advantage, on the one hand, of having been limited to patients with a certain diagnosis of migraine. On the other hand, migraine patients admitted to the emergency department were included in the study, an aspect that is still not sufficiently investigated in the literature, in our opinion.

## 5. Conclusions

In our study, migraine in children tends to have a favorable long-term prognosis at 10-year follow-up: 35% of patients experienced remission, and this percent of remission is in line with what is reported in the literature. To our knowledge, this was the first study conducted on selected pediatric patients at the time of admission to the emergency department. Therefore, due to the increasingly frequent use of the emergency department for the management of severe migraine attacks, further long-term follow-up studies conducted on pediatric patients admitted to the ED are needed, as this would allow a better understanding of the evolution of the disorder and, therefore, better therapeutic management. One may think that this category of patients might have a poorer long-term prognosis due to the intrinsic characteristics of migraine attacks. However, despite hospitalization at the ED, the percentage of remission of patients with migraine admitted to the emergency department substantially overlaps with the percentage of remission of children followed up in the clinic described in the literature.

We also underlined that in the present preliminary follow-up study, the family history of neuropsychiatric diseases, in particular, the presence of neurologic disorders in parents and sleep disturbances, are unfavorable long-term prognostic factors. Therefore, they are associated with a greater risk of migraine persistence over time. Future studies with larger samples, with several serial controls over time, are certainly needed. Furthermore, considering now that many of these subjects undergo MRI on admission to the ED, it might be interesting to repeat the acquisition if possible and assess the longitudinal MRI changes to better understand the observed clinical changes.

## Figures and Tables

**Table 1:** It shows the characteristics of the patients interviewed in the study.

Features	Patients n. 48
Average age of access to ED	8 years (IQR = 6–9)
Sex	
F	50% (24/48)
M	50% (24/48)
Neurologic disorders in the parents	57% (27/48)
Family history of migraine	40% (19/48)
Unphysiological course of pregnancy	33% (16/48)
Neuroradiological exams (CT, brain MRI)	62.5% (30/48)
Electrophysiological exams	52% (25/48)

Legend. ED: Emergency Department; CT: Computer Tomography; MRI: Magnetic Resonance Imaging.

**Table 2:** Comparison between migraine features of the sample in 2012 and 2022.

	Headache in 2012 n. 48	Headache in 2022 n. 31	*p*-Value (McNemar’s Test)
Frequency of attacks/month			0.057
≤2 attacks/month	52%	50%	
≥3 attacks/month	48%	50%	
Duration:			0.620
≤2h	58%	80.5%	
>2h	17%	19.5%	
Not remembered	25%		
Intensity:			0.038
1 (mild/moderate)	48%	71%	
2 (severe/unbearable)	52%	29%	
Aggravated by movement			1
YES	31%	51.6%	
NO	33%	48.4%	
Not remembered	35%	/	
Associated symptoms:			1
YES	66%	72%	
NO	32%	28%	
Not remembered	2%		

**Table 3:** Univariable logistic regression models of the long-term prognostic factors.

	Persistent Headache in 2022	Headache NOT Present in 2022	Odd Ratio (95% CI)	*p*-Value
	65% (31/48)	35% (17/48)		
Sex				
F	55% (17/31)	41% (7/17)	1.73 (0.53–5.94)	0.36
M	45% (14/31)	59% (10/17)		
Neurologic disorders in the parents				
YES	74% (23/31)	24% (4/17)	9.34 (2.53–41.64)	0.001
NO	26% (8/31)	76% (13/17)		
Physiological Pregnancy				
YES	65% (20/31)	71% (12/17)	0.88 (0.23–3.10)	0.83
NO	35% (11/31)	29% (5/17)		
Gestational Age				
≤37 GW	26% (8/31)	29% (5/17)	1.2 (0.30–4.44)	0.78
>38 GW	74% (23/31)	71% (12/17)		
Perinatal Suffering:				
YES	3% (1/31)	6% (1/17)	0.53 (0.02–14.09)	0.66
NO	97% (30/31)	94% (16/17)		
Social network hours/day:				
≤2 h/die	48% (15/31)	47% (8/17)	0.95 (0.29–3.12)	0.93
≥3 h/die	52% (16/31)	53% (9/17)		
Regular feeding:				
YES	58% (23/31)	82% (15/17)	0.30 (0.06–1.14)	0.09
NO	42% (8/31)	18% (2/17)		
Comorbidity:				
YES	23% (13/31)	12% (3/17)	2.19 (0.45–16.01)	0.36
NO	77% (18/31)	88% (14/17)		
Birth weight:				
≤2499 gr	26% (8/31)	11% (2/17)	0.38 (0.05–1.79)	0.26
≥2500 gr	74% (23/31)	89% (15/17)		
Games hours/day:				
≤2 h/die	48% (15/31)	47% (8/17)	1.44 (0.27–10.94)	0.68
≥3 h/die	52% (16/31)	53% (9/17)		
Sleep disorders:				
YES	45% (14/31)	6% (1/17)	13.18 (2.25–252.74)	0.018
NO	55% (17/31)	94% (16/17)		
Family history of migraine				
YES	48% (15/31)	24% (4/17)	3.05 (0.86–12.75)	0.09
NO	52% (16/31)	76% (13/17)		
Frequency of attacks/month in 2012				
≤2 attacks/month	35% (11/31)	18% (3/17)	8.48 (2.21–43.00)	0.003
≥3 attacks/month	65% (20/31)	82% (14/17)		
Duration in 2012:				
≤2 h	77% (20/26)	80% (8/10)	1.2 (0.22–9.37)	0.84
>2 h	23% (6/26)	20% (2/10)		
Intensity in 2012:				
1 (mild/moderate)	47% (14/30)	50% (8/16)	1.14 (0.34–3.90)	0.82
2 (severe/unbearable)	53% (16/30)	50% (8/16)		
Aggravated by movement in 2012				
YES	57% (12/21)	30% (3/10)	3.11 (0.66–17.75)	0.166
NO	43% (9/21)	70% (7/10)		
Associated symptoms in 2012				
YES	74% (20/27)	80% (12/15)	0.71 (0.13–3.13)	0.666
NO	26% (7/27)	20% (3/15)		

Baseline: Male, without parents with neurological disorders, no physiological pregnancy, >38 GW, no perinatal suffering, a social network for ≤2 h/die, regular feeding, no comorbidity, <2500 gr birth weight, a social network for ≤2 h/die, no sleep disorders, no family history of migraine, ≤2 frequency of attach/month in 2012, ≤2 h of duration in 2012, mild/moderate intensity in 2012, no aggravated migraine by movement in 2012, and no associated symptoms in 2012.

**Table 4:** Multivariable logistic regression models of the long-term prognostic factors.

	Odd Ratio (95% CI)	*p*-Value
Neurologic disorders in the parents		
YES	5.28 (1.21–25.83)	0.03
NO		
Frequency of attacks/month in 2012		
≤2 attacks/month	4.33 (0.9–24.26)	0.07
≥3 attacks/month		

**Table 5:** Main 10-year follow-up studies compared to our study.

Author’s Study	Population at Baseline	Population at Follow-Up	Years Follow-Up	Loss Rate (%)	Migraine Pz. at Follow-Up (%)	No Headache/Other Headaches Follow-Up (%)	Methods
Congdon 1979 [16]	300	208	10 ys	24%	71%	29%	Not specified
Dooley 1995 [15]	76	54	10 ys	29%	55%	45%	Phone interview
Hernandez-LaTorre 2000 [17]	181	181	10 ys	0	58.7%	41.3%	Visit
Brna 2005 [9]	45	45	10 ys	0	45%	55%	Phone interview
Monastero 2006 [12]	80	55	10 ys	31.7%	42%	58%	Phone interview
Galinski 2015 [9]	142	84	10 ys	40.9%	46%	54%	Phone interview
Manzo 2023	80	48	10 ys	40%	65%	35%	Phone interview/mail questionnaire

## Data Availability

All data are available to the authors following ethical restrictions.
